# The Impact of Tannic Acid Consumption on Bone Mineralization

**DOI:** 10.3390/metabo13101072

**Published:** 2023-10-12

**Authors:** Anna Winiarska-Mieczan, Siemowit Muszyński, Ewa Tomaszewska, Małgorzata Kwiecień, Janine Donaldson, Agnieszka Tomczyk-Warunek, Tomasz Blicharski

**Affiliations:** 1 Department of Bromatology and Food Physiology, Institute of Animal Nutrition and Bromatology, University of Life Sciences in Lublin, 20-950 Lublin, Poland; 2 Department of Biophysics, University of Life Sciences in Lublin, 20-950 Lublin, Poland; 3 Department of Animal Physiology, University of Life Sciences in Lublin, 20-950 Lublin, Poland; ewarst@interia.pl; 4 Department of Animal Nutrition, Institute of Animal Nutrition and Bromatology, University of Life Sciences in Lublin, 20-950 Lublin, Poland; malgorzata.kwiecien@up.lublin.pl; 5 School of Physiology, Faculty of Health Sciences, University of the Witwatersrand, Parktown, Johannesburg 2193, South Africa; janine.donaldson@wits.ac.za; 6 Laboratory of Locomotor System Research, Department of Rehabilitation and Physiotherapy, Medical University in Lublin, 20-090 Lublin, Poland; agnieszka.tomczyk-warunek@umlub.pl; 7 Department of Orthopaedics and Rehabilitation, Medical University in Lublin, 20-090 Lublin, Poland; blicharski@vp.pl

**Keywords:** tannic acid, bones, osteoporosis

## Abstract

Tannic acid (TA) is an organic compound belonging to the tannin group. Like other tannins, it has an affinity for endogenous proteins, including digestive enzymes, which can result in the reduced digestibility and absorption of nutrients. It can also form complexes with mineral components, reducing their absorption. In some cases, this can be beneficial, such as in the case of toxic metals, but sometimes it may have a detrimental effect on the body when it involves essential mineral components like Ca, P, Mg, Na, K, or Fe. Therefore, the impact of TA on bone health should be considered from both perspectives. This relatively short review summarizes the available information and research findings on TA, with a particular focus on its potential impact on bone health. It is worth noting that future research and clinical studies may provide more detailed and precise information on this topic, allowing for a better understanding of the role of TA in maintaining the integrity of the musculoskeletal system. Despite its brevity, this paper represents a valuable contribution to the analysis of the potential benefits and challenges associated with TA in the context of bone health. We anticipate that future research will continue along this important research line, expanding our knowledge of the influence of this compound on the skeletal system and its potential therapeutic applications.

## 1. Introduction

Osteoporosis is a chronic metabolic bone disease that gradually leads to a decrease in bone mass as well as structural abnormalities and decreased bone strength due to improper mineralization [1]. The initial stage is osteopenia, characterized by a reduction in bone mineralization, which can also be preceded by low peak bone mass [2]. Osteoporosis affects approximately 200 million people and is a significant global health concern [3]. The main factors contributing to osteoporosis include the age-related decline in estrogen levels, genetic factors, as well as dietary habits [4,5].

**Figure 1:**
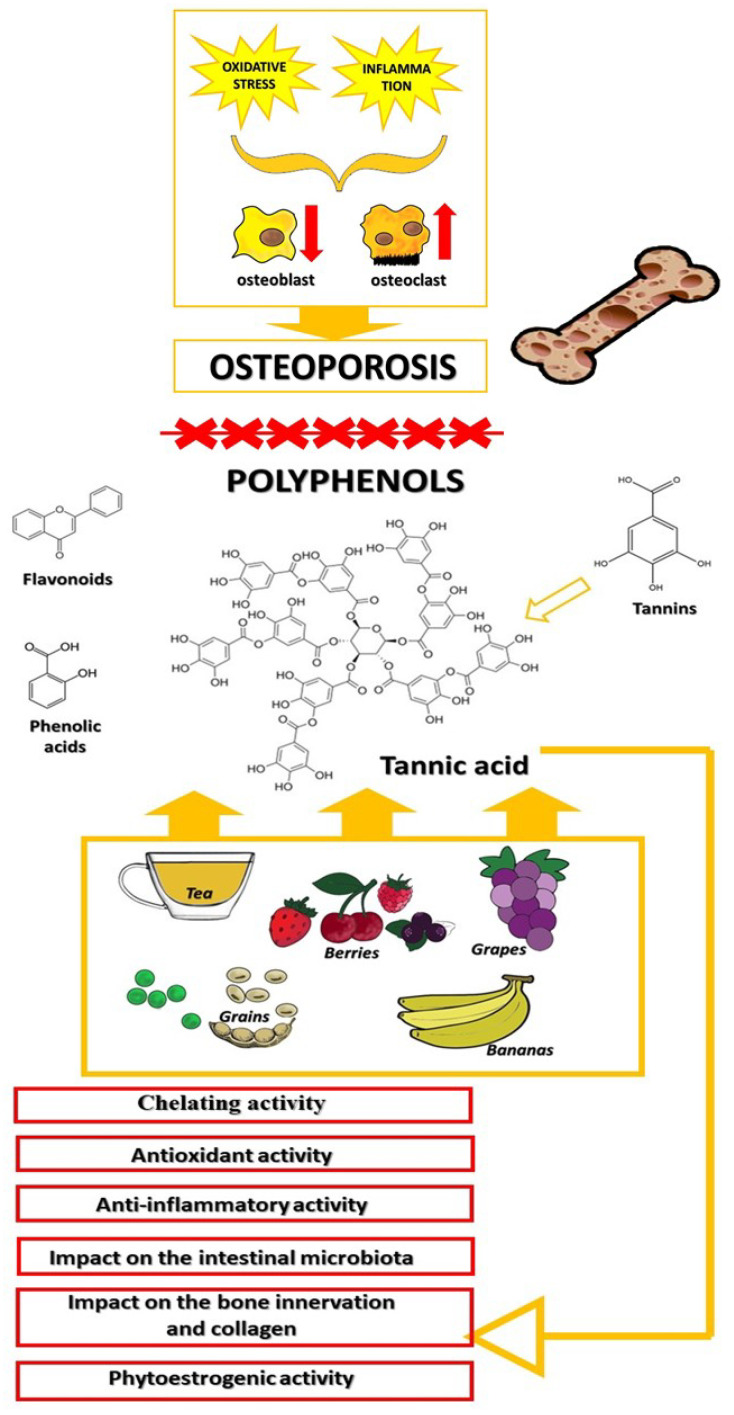
Classification and health benefits of polyphenols: the beneficial effects of consumption of food-derived polyphenols on bone health.

In osteoarticular diseases, especially osteoporosis, there is a strong correlation with inflammation and oxidative stress (an imbalance between oxidative and antioxidative processes) [6,7]. Under conditions of oxidative stress and inflammation, the activity and formation of osteoblasts are inhibited while the activity and formation of osteoclasts are promoted, which result in the further generation of reactive oxygen species (ROS) [6]. Oxidative stress, inflammation, and all their associated health complications, can be effectively mitigated through the use of exogenous antioxidants found in food, as demonstrated in numerous studies conducted on laboratory animals and humans (Figure 1).

Polyphenols, which are secondary metabolites in plants, are bioactive compounds that have a beneficial impact on humans and animals. In plants, phenolic compounds play a role in growth and reproduction, as well as provide protection against pathogens and herbivores [8]. Phenolic compounds include tannins, flavonoids, phenolic acids, and their chemically modified or polymerized derivatives [9]. Tannins are used in medicine, cosmetics, and nutrition, mainly due to their astringent, anticancer, and antimutagenic properties, which stem from their antioxidative and anti-inflammatory activities [10,11]. They also exhibit antimicrobial properties [12]. Tannic acid (TA) is an organic compound belonging to the tannin group. Like other tannins, it has an affinity for endogenous proteins, including digestive enzymes, which can result in the reduced digestibility and absorption of nutrients [13,14]. It can also form complexes with mineral components, reducing their absorption. In some cases, this can be beneficial, such as in the case of toxic metals [10], but sometimes it may have a detrimental effect on the body when it involves essential mineral components like Ca, P, Mg, Na, K, or Fe [15,16]. Therefore, the impact of TA on bone health should be considered from both perspectives.

TA exhibits strong anti-inflammatory and antioxidative effects [17]. Conversely, research has shown that chronic inflammation and oxidative stress are important markers of osteoporosis [6,7]. Therefore, it can be presumed that consuming TA should alleviate osteoporosis symptoms by improving the body’s condition. On the other hand, TA has chelating properties, particularly affecting mineral components crucial for bone mineralization, such as Ca and P [18]. In our review, we analyzed both the positive and negative effects of TA on bone health, citing available scientific research. Based on this, we have attempted to determine whether there are specific quantities of TA that could be harmful to bone health and whether individuals at risk of osteoporosis can consume TA in significant amounts.

This relatively short review summarizes the available information and research findings on TA, with a particular focus on its potential impact on bone health. It is worth noting that future research and clinical studies may provide more detailed and precise information on this topic, allowing for a better understanding of the role of TA in maintaining the integrity of the musculoskeletal system.

Despite its brevity, this paper represents a valuable contribution to the analysis of the potential benefits and challenges associated with TA in the context of bone health. We anticipate that future research will continue along this important research line, expanding our knowledge of the influence of this compound on the skeletal system and its potential therapeutic applications.

## 2. Methods Used to Search for Relevant Scientific Information

**Figure 2:**
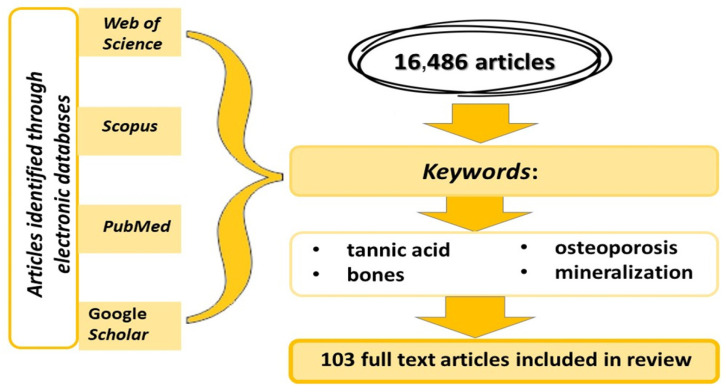
Research strategy employed in the review of the available literature.

An analysis of the information available in the global scientific literature was conducted in August and September 2023, utilizing databases such as PubMed, Scopus, Google Scholar, and Web of Science. The databases were searched for common and separate occurrences of the keywords “tannic acid”, “bones”, “osteoporosis”, and “mineralization” in both Polish and English languages. Ultimately, a total of 103 publications were utilized (Figure 2).

## 3. Bone Remodeling

Bone is a metabolically active organ that undergoes continuous remodeling processes to maintain its architecture, shape, and function throughout a person’s lifetime [19]. Bone remodeling is a process aimed at the continuous renewal of the human adult skeleton [20]. This process is characterized by a resorption phase lasting 30–40 days and a formation phase lasting approximately 150 days. Osteoclasts and osteoblasts are among the main key players in bone remodeling. Bone undergoes a four-stage remodeling cycle: (1) the quiescent phase, during which osteocytes activate bone lining cells, which, in turn, initiates osteoclast differentiation leading to bone surface exposure; (2) the activation phase, in which osteoclasts resorb the exposed bone, subsequently undergoing apoptosis; (3) the reversal phase, which involves the migration of macrophage-like cells to the resorbed site, followed by debris clearance and the release of stimulating factors; (4) the formation phase, which ends the process, during which matrix mineralization occurs while osteoblasts either undergo apoptosis or become embedded in the bone matrix as osteocytes [21].

Osteoclasts originate from hematopoietic progenitors as precursors and are subsequently attracted to resorption sites through chemotactic factors, including parathyroid hormone (PTH), tumor necrosis factor alpha (TNFα), and prostaglandin E2 (PGE2) [20]. These chemotactic factors increase the expression of the receptor activator of NF-κB ligand (RANKL), which binds to the RANK receptor on osteoclast precursors, leading to their fusion and the formation of mature osteoclasts [22]. RANKL is a TNF-related ligand present on the surface of osteoblasts [20]. Bone marrow mesenchymal stem cells are a source of progenitor cells for osteoblasts. Osteocytes, on the other hand, are cells derived from osteoblasts that become embedded in the mineralized bone matrix and play a role in maintaining the bone matrix [23]. The attraction of preosteoclasts to remodeling sites is chemotactically controlled by osteoblasts. In response to inflammatory factors, macrophage colony-stimulating factor (M-CSF) and monocyte chemoattractant protein 1 (MCP-1) are released, which attract osteoclast precursors to the area [20]. Proteases released by resorbing osteoclasts activate transforming growth factor-beta 1 (TGF-β1) (which, in turn, attracts osteoblasts and enhances proliferation, differentiation, proteoglycan synthesis, and type I collagen production) and stimulate type II collagen expression and proteoglycan synthesis by chondrocyte precursor cells [24].

Research indicates that bioactive food components, e.g., phenolic compounds, can positively or negatively influence bone remodeling processes.

## 4. Tannic Acid

**Figure 3:**
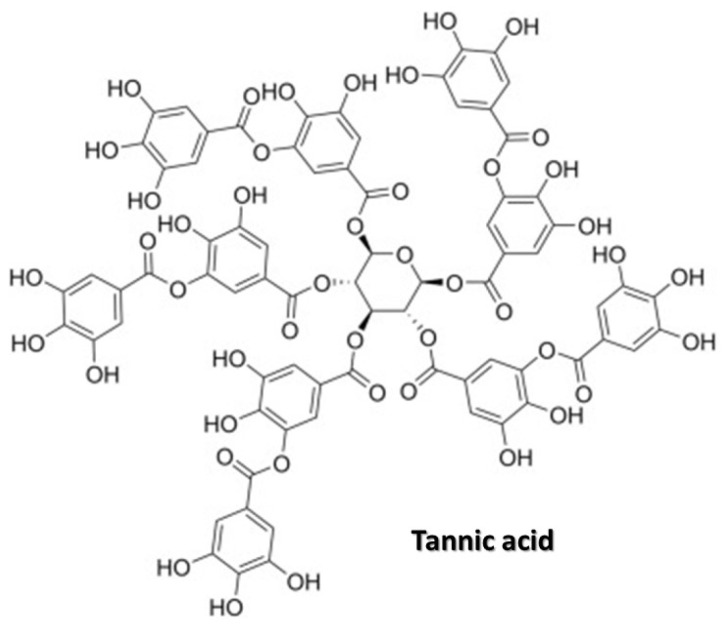
Chemical structure of tannic acid.

Tannic acid (TA) is an ester of gallic acid and digallic acid with a centrally located glucose molecule (Figure 3). In the plant world, tannins protect tissues from insect attacks and fungal pathogens [17]. TA is found in many herbaceous and woody plants, including legumes, sorghum, beans, bananas, raspberries, grapes, and teas [25]. TA is a naturally occurring polyphenolic antioxidant. Over the last decade, it has gained popularity in biomedical research due to its biochemical properties, stemming from the presence of numerous hydroxyl groups and its ability to form hydrogen bonds with biomolecules [17]. It exhibits anti-inflammatory, antioxidative, bactericidal, antiviral, and antifungal properties [17]. At the same time, it has a strong chelating effect on mineral components [10], which means that excessive consumption can influence bone mineralization and contribute to an increased risk of osteoporosis.

## 5. Influence of Tannic Acid on Bone Mineralization and Structure

### 5.1. Chelating Activity of Tannic Acid

In condensed tannins, the primary group involved in metal ion complexation is the ortho-dihydroxyl group located on the flavonoid B-ring [26]. A study conducted by Jaramillo et al. [16] on a group of 28 adult women demonstrated that calcium ions (Ca) readily bind to the hydroxyl groups of TA. Consequently, the presence of TA in the diet may limit the availability of calcium (Ca) and have an adverse effect on bone mineralization.

Furthermore, excessive consumption of TA can also impact the absorption of other important mineral components, such as phosphorus (P), magnesium (Mg), sodium (Na), potassium (K), and iron (Fe), which may negatively affect overall bone health [10,15]. Therefore, individuals at risk of osteoporosis or those with limited intake of these minerals in their diet should be aware of the detrimental effects of excessive TA consumption on bone health. It is advisable to maintain a balanced diet and avoid excessive TA intake to ensure good bone health.

However, some studies suggest that the chelating properties of TA may have a protective effect on bones. TA has strong chelating properties, which means it can bind to and neutralize heavy metals like cadmium (Cd) and lead (Pb). When these toxic metals accumulate in bone tissue, they can have harmful effects on bone health. TA’s ability to chelate these metals may help protect bones from their detrimental impact [27].

Research was conducted to evaluate the effect of administering TA solutions in drinking water at concentrations of 0.5%, 1.0%, 1.5%, 2.0%, or 2.5% to growing and adult rats exposed to cadmium (Cd, 7 mg/kg diet) and lead (Pb, 50 mg/kg diet) for 6 weeks. These concentrations corresponded to a daily TA intake of approximately 0.10–0.45 g/day [28,29]. These studies evaluated the structure and properties of bones, as well as the morphology of the articular cartilage and the growth plate. TA led to improvements in the histomorphometric parameters of trabecular bone and the articular cartilage structure, which are primarily attributed to the chelating action of TA on Cd and Pb [10]. It is conceivable that TA has a greater affinity for Cd and Pb than for calcium (Ca), as evidenced by studies showing increased Ca content in the bones of rats receiving higher doses of TA (2.0% and 2.5%) compared with the control group (without TA) [29]. TA treatment appears to mitigate some of the adverse effects of Cd and Pb exposure on bone health in rats. It reduces Pb content in bones, maintains bone Ca levels, promotes bone elongation, and enhances bone strength and structure. Additionally, TA treatment seems to have a positive impact on the articular cartilage, contributing to its integrity. However, it is worth noting that the effectiveness of TA may vary depending on its concentration [28,29]. Therefore, the chelating properties of TA can be advantageous in cases of heavy metal poisoning, positively impacting bone mineralization in such scenarios. The impact of TA on bone mineralization can vary depending on individual factors such as the amount of TA consumed, age, gender, and overall health. In summary, TA can have both positive and negative effects on bone mineralization, depending on the context and the amount of TA consumed. Therefore, it is important to consume substances containing TA in moderation.

### 5.2. Antioxidant Activity of Tannic Acid

Phenolic compounds exhibit antioxidant properties through (1) limiting the production of ROS by chelating trace elements and inhibiting oxidative enzyme activity; (2) scavenging reactive oxygen species (ROS); (3) donating electrons or hydrogen atoms, facilitating the neutralization of singlet oxygen; and (4) increasing the activity of endogenous antioxidants [30]. Oxidative stress affects bone mineral density by (1) increasing the rate of osteoclastogenesis; (2) reducing the differentiation rate of osteoprogenitors into osteoblasts; and (3) decreasing osteoblast activity and/or increasing osteoblast and osteocyte apoptosis [31]. Phenolic compounds often work synergistically with other antioxidants and vitamins (e.g., vitamins: C, D, E, and vitamin K) to conserve bone health. These synergistic effects can enhance the overall antioxidant defense system and minimize oxidative damage to bones [32].

Oxidative stress is considered a significant pathogenetic factor in osteoporosis. Excessive levels of ROS induce the apoptosis of osteocytes, which, in turn, affects the synthesis of factors responsible for bone formation processes, such as RANKL, sclerostin, and fibroblast growth factor 23, leading to impaired bone remodeling and increased bone resorption [33]. The heightened osteoclastogenesis observed under conditions of oxidative stress is a result of increased RANKL activity and decreased osteoprotegerin activity [31]. On the other hand, endogenous antioxidants produced by osteoblasts, such as glutathione peroxidase (GPX), counteract the detrimental effects of ROS on bone [34].

Studies conducted on rats have shown that the oral administration of TA in aqueous solutions (0.5–2.5%, which corresponded to a daily TA intake of 0.10–0.45 g) enhances the antioxidant potential of rats exposed to cadmium (Cd) and/or lead (Pb) [10,35]. The 2.0% solution was found to be the most effective. Similarly, favorable effects were observed in rats poisoned with Cd and Pb, to which black, green, white, and red tea solutions containing 2.0% TA were administered [10,36].

A TA-rich extract from grape seeds reduces ROS levels and the production of peroxides, while simultaneously increasing the expression of antioxidant enzyme genes [37]. The use of exogenous antioxidants increases bone density by reducing the activity of RANKL, as demonstrated in animal studies [38]. In an in vitro study, the impact of a polyphenol-rich grape seed extract on the osteogenic differentiation of human mesenchymal stem cells was examined [39]. It was observed that this extract led to a reduction in the RANKL/osteoprotegerin ratio and increased the expression of genes involved in osteoblast differentiation. Some studies suggest that phenolic compounds stimulate osteoblast differentiation and activity, promoting bone formation. This is achieved through the upregulation of specific genes and proteins involved in osteogenesis [40].

### 5.3. Anti-Inflammatory Activity of Tannic Acid

Inflammatory conditions, characterized by elevated levels of pro-inflammatory interleukins (IL-1, IL-6, TNF-α), can inhibit osteoblast activity by suppressing the expression of Runt-related transcription factor 2 (RUNX2), which is responsible for osteoblast differentiation, and stimulate the synthesis of RANKL, which, in turn, activates osteoclasts [6]. This means that inflammation directs the changes occurring in bones toward resorption, promoting bone resorption processes [20]. In addition to RUNX2 and RANKL, chemical mediators of inflammation include TNFα, glucocorticoids, histamine, bradykinin, PGE2, systemic RANKL from immune cells, IL-1, and IL-6 [20]. In vitro studies have shown that inflammatory macrophages interrupt the maturation and mineralization of osteocytes by regulating the Notch signaling pathway [13]. The Notch signaling pathway is a canonical signaling pathway involved in various biological processes. It plays a significant role in osteocyte maturation, as suppressing Notch in osteocytes prevents them from reaching maturity [41]. Pathological bone resorption has been documented in at least 100 chronic inflammatory conditions, such as rheumatoid arthritis, periodontal disease, and Crohn’s disease [19].

Polyphenols reduce inflammation by suppressing pro-inflammatory cytokines, inhibiting TNF-α, and inducing apoptosis, which lead to a reduced risk of DNA damage [42]. TA possesses anti-inflammatory properties by reducing the production of pro-inflammatory cytokines, and this anti-inflammatory effect can indirectly support bone health. Polyphenols also exhibit immunomodulatory effects by inhibiting the proliferation of autoimmune T cells and reducing the levels of pro-inflammatory cytokines [42]. A study of polyphenols from pomegranate peels showed a reduction in the levels of pro-inflammatory cytokines (TNF-α, IL-1β, IL-6) and other inflammatory mediators through a reduction in the expression of inducible nitric oxide synthase and cyclooxygenase-2 [43]. It has been demonstrated that flavonoids stimulate bone defect regeneration, minimize inflammation, and have a significant anticatabolic effect by reducing trabecular bone loss and increasing bone mineral density [44]. Research conducted on Cobb 500 chicks with necrotic enteritis showed that dietary supplementation of TA (250, 500, 750, and 1000 mg/kg) resulted in decreased TNF-α levels and increased levels of anti-inflammatory parameters (T-AOC, IL-4, and IL-10) in serum [11]. Similarly, favorable effects were observed in studies on rats with induced inflammation that were given TA at doses of 25 or 50 mg/kg [45]. The anti-inflammatory action of TA was also observed in lipopolysaccharide-induced BV2 microglial cells, where it inhibited NF-κB activation and reduced the levels of IL-6, IL-1β, and TNF-α [46]. Moreover, TA can influence various signaling pathways related to bone remodeling. For example, it can affect the Wnt/β-catenin pathway, which plays a crucial role in bone formation and resorption. By modulating these pathways, phenolic compounds can impact bone metabolism positively [47,48].

### 5.4. The Relationship between Tannic Acid, the Gut Microbiota, and Bones

Previous studies have shown that there is an inseparable connection between the gut microbiota and bone homeostasis. Therefore, disruptions to the structure and in the functioning of the microbiota may play an initiating and reinforcing role in disturbing bone metabolism during the development of osteoporosis [49]. The gut microbiota influences skeletal homeostasis by affecting the host’s metabolism, immune function, hormone secretion, and the gut–brain axis [50]. It has been demonstrated that gut microbiological dysbiosis is a risk factor for bone-related diseases in humans [51]. The gut flora is highly diverse, and the exact number of species is not fully known, with an estimated 1500 bacterial species residing in the human gastrointestinal tract [52]. In healthy adults, the structure of the microbiota in different segments of the gastrointestinal tract is primarily influenced by the pH of the environment and the presence of oxygen. Depending on the gut segment, *Helicobacter pylori*, *Lactobacillus*, *Streptococcus*, *Candida albicans*, *Bacteroides*, *Clostridium*, *Enterococcus*, *Veillonella*, *Firmicutes*, *Bacteroidetes*, *Proteobacteria*, and *Actinobacteria* may predominate [53].

Research by Wang et al. [54] revealed that individuals with osteopenia exhibited an increased abundance of *Firmicutes* and a reduced number of *Bacteroidetes* compared with the control group. Moreover, *Synergistetes* were present in both osteoporotic and osteopenic patients but were absent in healthy individuals. Bacteria from the genera *Lachnoclostridium* and *Klebsiella* were found in significant quantities in individuals with osteoporosis and osteopenia but were less prevalent in healthy subjects. Furthermore, an improvement in bone mineral density was observed through the inhibition of the inflammatory process when using probiotics with immunomodulatory activity toward IGF-1, TNF-α, and IL-1β [55]. The influence of gut bacteria on changes in bone mass has also been identified, suggesting a causative link between the microbiota and bone development [56]. Research results also clearly indicate that the gut flora plays a significant role in osteoclastogenesis and bone healing processes, especially in the case of osteoporosis [57]. This is largely associated with the fact that the gut microbiota modulates the absorption of mineral components and the production of vitamins essential for bone homeostasis [58]. The gut microbiota enhances calcium absorption through the production of short-chain fatty acids (SCFAs), which are generated during the fermentation of dietary fiber by symbiotic bacteria such as *Bifidobacterium* [58]. In vivo studies have shown that increased SCFA levels are positively correlated with increased bone mass and prevent bone loss [59]. Additionally, the high SCFA content in the intestines lowers the pH in the gut lumen, enhancing the solubility of mineral compounds and inhibiting the formation of calcium–phosphate complexes, thereby increasing calcium availability and absorption [59]. SCFAs also increase paracellular calcium transport across the intestinal epithelium [60]. Consequently, individuals with low levels of SCFA-producing *Clostridium cluster* XIVa and *Lachnospiraceae* in their microbiota exhibit a lower BMD and a higher risk of fractures [58]. Gut microbiota can also modulate vitamin D metabolism through secondary bile acids [58].

The mucous membrane of the gastrointestinal tract serves a protective function against external factors [61]. In response to antigens present in the gut content, it stimulates the secretion of immunoglobulins and activates macrophage, lymphocyte, and cytokine responses. The gastrointestinal epithelium acts as a transport barrier, controlling the absorption, uptake, and secretion of various substances. Disruptions in the structure of the gut microbiota can be a cause of abnormal gut construction. Phenolic compounds influence the growth of gut bacteria by inhibiting the activity of extracellular microbial enzymes, depriving microorganisms of substrates essential for their growth or affecting bacterial metabolism [61]. They can stimulate the growth of beneficial bacterial species or inhibit the development of harmful ones. In turn, the microbiome can metabolize phenolic compounds and produce bioactive molecules [62,63]. The use of TA in the form of microcapsules resulted in improved duodenal morphology, intestinal nutrient transport, and gut microbiota composition compared with the control group [64]. Microbiological analysis of the cecum in Cobb 500 broilers with necrotic enteritis showed that TA administration improved gut barrier function and regulated gut flora. Moreover, the decreased levels of D-lactic acid and diamine oxidase in the serum indicate the potential positive effect of TA in maintaining gut barrier integrity [11].

However, some studies suggest a reduction in bone mineralization due to the use of TA. In a study by Choi et al. [65], broiler chickens receiving TA at doses ranging from 0.5 to 2.5 g/kg exhibited a linear decrease in BMD in their bones, despite improvements in the microbiota profile. Similar bone-related results were reported by other authors [66,67]. These findings are likely due to the fact that tannins inhibit the utilization of calcium, phosphorus, and iron through chelation, which may negatively affect bone development in animals. It is worth noting, however, that the reduction in bone mineralization observed in these studies depended on the quantity of TA in the diet, suggesting that the issue may not be the TA itself but rather its quantity. This topic requires further investigation.

### 5.5. Tannic Acid as a Biomaterial in Bone Treatment

Due to its antibacterial and anti-inflammatory properties, TA is also considered as a component in various types of prostheses and biomaterials for the treatment of bone defects. Materials used in the production of orthopedic implants can lead to the generation of ROS in the body, primarily at the implant’s surface, which hinders bone growth and may result in poor osseointegration at the implant interface. Such complications have been observed, for example, with implants made from titanium alloys [40]. Oxidative stress can significantly impair the biological function of osteoblasts, thereby impeding early bone formation and even causing implant failure [68]. Due to its strong antioxidant, anti-inflammatory and antibacterial properties, TA has the potential to become a highly suitable coating for orthopedic implants. This was confirmed in a study by Buyn et al. [69], where TA-containing nanoparticles were used in the regeneration of damaged bones in mice. Moreover, a study was conducted using a crosslinked TA complex and collagen fibers to induce dentin biomineralization [70]. In this study, complete dentin remineralization was achieved within 4 days, and improvements in the mechanical and antienzymatic properties of dentin were also observed.

### 5.6. Tannic Acid and Bone Innervation

Nerves in bone play a crucial role in integrating central signals related to vascular tone and pain with the regulation of skeletal cells like osteoblasts and osteoclasts. These nerves utilize neurotransmitters to control various aspects of skeletal homeostasis, even though true synapses have not been found in bone. Instead, neurotransmitters are released from nerve fibers into the extracellular space and diffuse to target-cell receptors, allowing for the simultaneous regulation of multiple skeletal functions. Bone innervation pertains to the presence of nerve fibers and neurons within the bone tissue, contributing to pain perception, bone remodeling, and other physiological processes [71,72]. Various types of nerve fibers have been identified within the bone tissue, including peptidergic sensory fibers and autonomic nerve fibers, which interact with osteoblasts, osteoclasts, hematopoietic cells, and blood vessels in the bone marrow [73]. The sympathetic nervous system (SNS), operating through adrenergic receptors, plays a crucial role in the central control of bone formation. A noteworthy study involving dopamine-ß-hydroxylase (DBH) deficiency shed light on the significance of the SNS in bone physiology. DBH is essential for the production of norepinephrine and epinephrine, catecholamines that bind to adrenergic receptors. Surprisingly, DBH-deficient mice exhibited increased bone mass [74,75].

Key neurotransmitters implicated in bone regulation encompass the following: (1) calcitonin gene-related peptide (CGRP): This sensory neuropeptide exhibits osteoanabolic effects by enhancing osteoblast proliferation, reducing the apoptosis of osteoprogenitors, stimulating osteogenic gene expression, and indirectly suppressing osteoclast maturation and activity [76]; (2) Substance P: This neuropeptide plays a role in vasodilation, inflammation, and pain. It promotes both osteoblast and osteoclast activity as well as osteoclastogenesis; however, its effects on bone can be multifaceted due to its involvement in inflammation [76]; (3) Norepinephrine: Released by sympathetic nerves, norepinephrine can negatively impact bone by suppressing osteoblast proliferation and promoting osteoclastogenesis through β2-adrenergic receptors. Nevertheless, it can also positively regulate hematopoiesis and influence bone metastasis [77]; (4) Neuropeptide Y: This neuropeptide affects bone metabolism centrally and peripherally; central control involves the inhibition of Y2-expressing neurons, while local control impacts osteoblasts through Y1 receptors, increasing bone formation [77]; (5) Acetylcholine: Primarily released by cholinergic nerves, acetylcholine plays regulatory roles in bone metabolism by interacting with receptors on bone lineage cells. it influences the degree of proliferation, differentiation, and ossification within bone tissue, potentially affecting bone mass [78]; (6) Vasoactive intestinal peptide (VIP): Co-released with acetylcholine from cholinergic nerve terminals, VIP acts on VIP receptors, VPAC1 and VPAC2. VIP stimulates osteoblasts, increases cAMP production, reduces osteoclast production, promotes osteogenic differentiation of bone cells, and stimulates angiogenesis [79,80].

These neurotransmitters exert their effects on bone by binding to specific receptors, modulating second messenger signaling cascades, and influencing various cellular responses. These neural signals are critical for maintaining skeletal homeostasis. Furthermore, sensory denervation consistently reduces osteoclastic activities, while sympathectomy often increases them, especially under stressful conditions [81]. Although TA has shown neuroprotective effects due to its potent anti-inflammatory and antioxidant properties [82,83], research on TA’s specific impact on bone innervation remains limited. TA has been explored for its impact on bone health, particularly concerning bone density, mineralization, and inflammatory processes, but its direct influence on bone innervation warrants further investigation. TA’s neuroprotective properties may indirectly benefit nerve integrity and function within bone [84,85]. To gain a comprehensive understanding of the relationship between TA and bone innervation, future research should investigate whether TA directly impacts nerve fibers and neurons within the bone tissue and how it may affect pain perception, sensory functions, or other aspects of bone innervation.

### 5.7. Tannic Acid and Collagen

There are 29 variations of collagen proteins which exist, each of which exhibit slight differences in both their functional and structural characteristics [86]. The most well-known forms of collagen include the following: type I collagen (found in skin, bones, and tendons), type II collagen (present in cartilage), and type III collagen (associated with vascularization). Collagen is a critical component of bone, providing the structural framework for bone strength. Collagen fibers in bone tissue are primarily composed of type I collagen, which forms a dense network and gives bones their tensile strength [87].

Phenolic compounds found in certain foods and beverages have been studied for their potential role in supporting collagen stability in bones [88,89]. These compounds are known for their antioxidant properties, which can help protect collagen fibers from oxidative damage. Oxidative stress can lead to the degradation of collagen and other important components of the bone matrix, potentially weakening the bone structure [90]. By reducing oxidative stress and providing antioxidant support, phenolic compounds may contribute to the preservation of collagen integrity in bones [91]. This, in turn, can have a positive impact on bone strength and resilience, helping to maintain overall bone health.

The introduction of TA to collagen during the cross-linking process does not impact the integrity of collagen’s triple-helical structure [92]. TA has been extensively researched for its ability to modify collagen since it interacts with the hydrophilic functional groups found in polymeric chains, including both amino and carboxylic groups [93]. When comparing collagen-based materials cross-linked using starch dialdehyde and TA, it was observed that the addition of TA resulted in better biocompatibility [94]. This suggests that naturally derived compounds proposed as cross-linkers may be more suitable for biomedical applications [91].

### 5.8. Tannic Acid and Bone Loss in Menopause

Phenolic compounds may mitigate the impact of risk factors for osteoporosis, such as hormonal changes during menopause. Studies on the effects of tannic acid (TA) in postmenopausal women with osteoporosis is still ongoing, but there are promising results and potential benefits associated with its use. Inflammatory conditions and oxidative stress are strongly correlated with musculoskeletal diseases, particularly osteoporosis [6,7]. TA, due to its potent antioxidant and anti-inflammatory properties, may have the potential to protect the bones of postmenopausal women, who are more susceptible to bone loss.

During menopause, estrogen production decreases, which can lead to accelerated bone loss and an increased risk of osteoporosis. There is research indicating that TA affects estrogen receptor expression in women with estrogen-dependent tumors [95]. Therefore, it can be speculated that by regulating estrogen secretion, TA may have a protective effect on other conditions related to estrogen levels in the body, including osteoporosis. Booth et al. [95] demonstrated that the presence of TA inhibited proliferation and induced apoptosis (through the activation of caspase 3/7 and caspase 9) in estrogen-dependent MCF7 breast cancer cells. Conversely, Nie et al. [96] did not observe any impact of TA on MCF7 breast cancer cells. However, these authors noted that TA inhibits fatty acid synthase activity, an enzyme that is overexpressed in human breast cancer cells, which could be one of the possible ways to induce apoptosis in these cells. The authors suggested that perhaps different results would be obtained if a higher concentration of TA were used in the experiment. This is in line with the observations of Ngobili et al. [97] and Jordan and Booth [98], who noticed that in estrogen-receptor-positive breast cancer cells (ER+), TA induced concentration-dependent apoptosis.

In studies where breast cancer cells were exposed to tamoxifen and/or tannin nanoparticle extract, it was found that tannins enhance the action of tamoxifen and induce cell proliferation and apoptosis, as indicated by an increase in DNA fragmentation [99].

## 6. Summary

**Figure 4:**
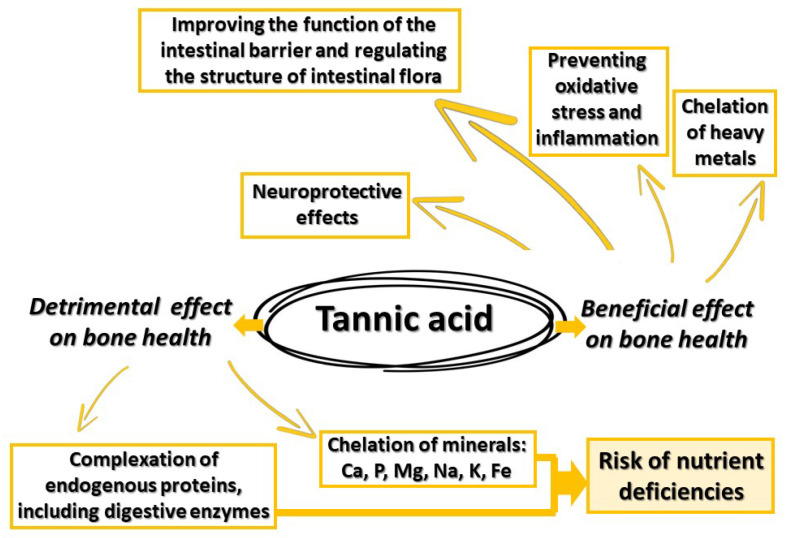
Detrimental and beneficial effects of TA on bone health.

This review stands out for its moderate length, which is understandable given the specificity of the research on TA. It is important to note that previous studies have mainly focused on the properties of this compound in contexts other than bone metabolism. Therefore, even though TA exhibits fascinating bioactive properties, its potential impact on bones remains largely unexplored. Research on TA has predominantly concentrated on its antioxidant and anti-inflammatory properties, as well as its impact on overall health. Only recently has the interest of researchers turned toward its potential influence on bone health. The results of these studies seem promising, suggesting that TA may have both positive and negative effects on bone health, depending on the context and dosage (Figure 4).

This is an area that deserves further investigation and experimentation to better understand how TA may affect bones, particularly in the context of osteoporosis and other bone-related conditions. Additionally, it is important to consider a balanced approach to TA consumption to avoid excessive intake, which could have adverse effects on bone health. Therefore, while there is a wealth of promising data regarding the potential health benefits of tannic acid, further research is necessary to precisely elucidate the mechanisms of its impact on bones and to establish optimal dosages and consumption conditions in the context of bone health. Future studies may contribute to developing more precise recommendations for tannic acid intake to maintain healthy bones.

Osteoporosis is a chronic metabolic bone disease characterized by a gradual decrease in bone mass and structural abnormalities, resulting in decreased bone strength. Inflammation and oxidative stress play a significant role in osteoporosis development. TA, a type of polyphenol, has both positive and negative effects on bone health. Its chelating properties can hinder the absorption of essential minerals like calcium and phosphorus, which may negatively impact bone mineralization. However, TA’s chelating ability also helps remove toxic heavy metals like cadmium and lead, potentially protecting bones from their harmful effects. TA exhibits antioxidant and anti-inflammatory properties, which can counteract the oxidative stress and inflammation associated with osteoporosis. It may also modulate signaling pathways involved in bone remodeling. However, the impact of TA on bone health varies depending on factors like its concentration and the patient’s age, gender, and overall health. The gut microbiota plays a crucial role in bone homeostasis, and TA may influence the gut microbiota–bone relationship. TA can affect the composition of gut bacteria, potentially influencing the absorption of minerals and vitamins essential for bone health. TA has potential as a biomaterial for orthopedic implants due to its antibacterial and anti-inflammatory properties. It may help improve osseointegration and reduce oxidative-stress-related complications associated with implants. The role of TA in bone innervation remains understudied, but its neuroprotective properties suggest potential indirect benefits for bone innervation and pain perception. TA may help preserve collagen integrity in bones by protecting collagen fibers from oxidative damage. Collagen is critical for bone strength. In postmenopausal women, TA’s antioxidant and anti-inflammatory properties may offer protection against bone loss, especially in the context of estrogen deficiency. Further research is needed to fully understand TA’s effects on estrogen-related conditions.

Despite TA’s positive impact on bone health and overall bodily functions, it is important to consider the doses applied. TA only benefits the body when its concentration in food is not too high. TA contains active hydroxyl and carboxyl groups that can form complexes with proteins, polysaccharides, alkaloids, and nucleic acids [29,100]. These properties can impede the digestion and absorption of nutrients. According to EFSA [101], using TA in amounts of up to 15 mg/kg of feed is safe for all animal species. However, the maximum safe TA intake for humans has not been established, although it is generally recommended that people should not exceed 560 mg of TA per day [29]. In vitro research has suggested potential mutagenic effects of tannic acid, but these effects have not been confirmed in in vivo studies [101]. Additionally, mild necrotic changes in the livers of rats were observed when they were given a 0.4% aqueous TA solution, despite TA not affecting overall bodily functions or blood parameters [102]. On a different note, TA has been found to inhibit histone acetyltransferase and to prevent non-alcoholic fatty liver disease in both in vivo and in vitro studies [103].

## 7. Perspectives

Future research should focus on determining the optimal dosage of TA for bone health. This includes investigating the concentration that provides the most benefits without negatively impacting mineral absorption. Evaluating the potential of TA as a dietary supplement for individuals at risk of osteoporosis, especially postmenopausal women, can provide valuable insights into its clinical applications. Further research on the ability of TA to preserve collagen integrity in bones may have implications for preventing bone fragility. Investigating TA’s potential in managing hormone-related conditions, such as those involving estrogen receptors, can expand its therapeutic applications. Investigating the development of TA-based biomaterials for orthopedic implants and bone regeneration could offer innovative solutions in the field of bone surgery. Exploring TA’s direct impact on bone innervation, pain perception, and sensory functions can shed light on its role in overall bone health. Understanding the complex interaction between TA and the gut microbiota, including its effects on mineral absorption and vitamin metabolism, can provide insights into the microbiota–bone relationship.

In summary, tannic acid’s multifaceted effects on bone health warrant continued research to harness its potential benefits while addressing its limitations. Its role in mitigating oxidative stress, inflammation, and interactions with the gut microbiota make it a promising candidate for osteoporosis management and orthopedic applications.
